# Endodontic Management of Maxillary Central Incisor with Rare Root Canal Anatomy

**DOI:** 10.7759/cureus.7851

**Published:** 2020-04-27

**Authors:** Sudha Yadav, Ruchika R Nawal, Sangeeta Talwar

**Affiliations:** 1 Conservative Dentistry and Endodontics, Maulana Azad Institute of Dental Sciences, New Delhi, IND

**Keywords:** maxillary central incisor, type v morphology, root canal

## Abstract

A maxillary central incisor presenting with more than one root or root canal is an exceptionally rare scenario considering the fact that most of the anatomic studies describe maxillary central incisor as a single rooted tooth with single canal. However, several case reports have shown the presence of up to four canals in maxillary central incisors. The aim of this article is to present a case report of maxillary central incisor with a rare anatomic variation, i.e. Vertucci’s type V root canal anatomy. Failure to locate and clean additional root canal system may lead to post treatment disease. Hence, an astute clinician should be aware of possible anatomic variations this tooth might present with.

## Introduction

Root canal anatomy is highly variable and complex. It has been studied using several techniques such as clearing, histological sectioning, microscopic/stereomicroscopic evaluation and ground sections; it provides valuable information in understanding complex internal root canal anatomy [1-6]. Maxillary central incisors are described in the literature as single rooted teeth with single root canal. Vertucci in 1984 studied the root canal anatomy of human permanent teeth using the clearing technique and concluded that 100% maxillary central incisors had a single root canal [7]. However, there is no dearth of case reports showing variation in the root canal anatomy of maxillary central incisors [8-11]. Most of these case reports illustrate maxillary incisor with two roots and two canals. The aim of this article is to present a case report describing non-surgical endodontic management of a maxillary central incisor with Vertucci’s type V morphology.

## Case presentation

A 65-year-old female from Delhi, India (nationality: Indian; ethnicity: South Asian) reported to Department of Conservative Dentistry and Endodontics at Maulana Azad Institute of Dental Sciences in India with the complaint of pain in the upper front tooth. Medical history was insignificant. Root canal treatment was started by a private practitioner for tooth number 21 one week back. But the patient did not find any relief in pain.

**Figure 1:**
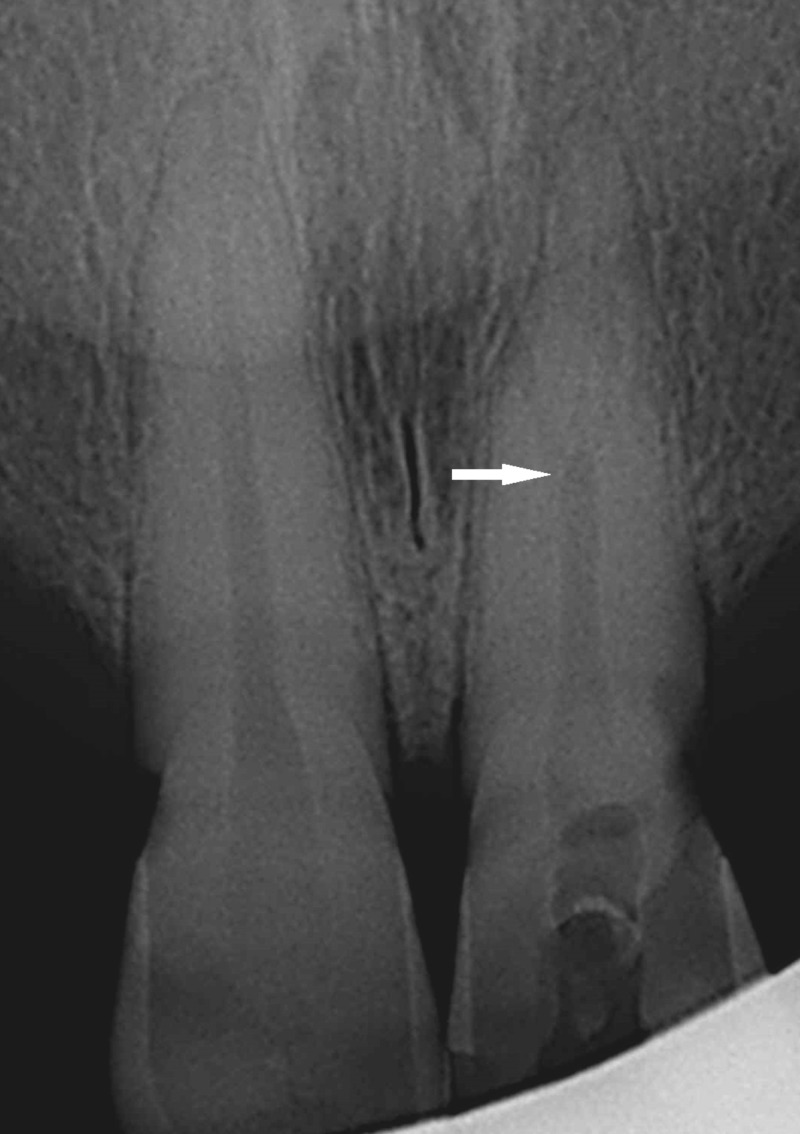
Preoperative periapical radiograph of tooth number 21 showing sudden break in root canal outline in the apical third (shown with arrow).

On clinical examination, tooth number 21 was discolored, tender on percussion and the temporary restoration was missing. The external morphology of the crown was normal. Periodontal findings were within normal limits. Preoperative radiograph showed a single rooted left maxillary central incisor with one main canal bifurcating at apical third (Figure 1). Periapical periodontal ligament widening was also evident. A diagnosis of previously initiated therapy with symptomatic apical periodontitis was made for tooth number 21. Although the patient was inclined towards extraction of the tooth due to severe pain, she was advised to undergo completion of endodontic treatment. Informed consent was obtained from the patient after thorough discussion regarding the risks, treatment plan and outcome of the treatment.

**Figure 2:**
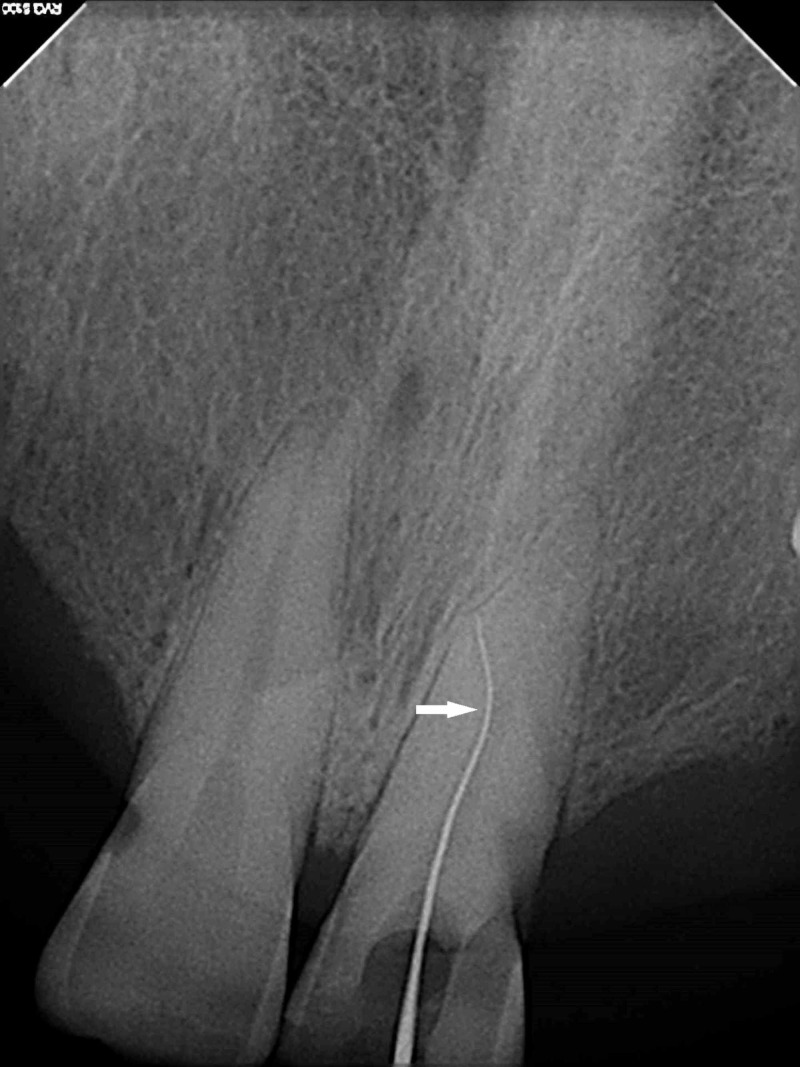
Negotiation of accessory canal (marked with arrow) using a #10 K-file.

Access opening was modified after rubber dam isolation under a dental operating microscope (DOM) (Carl Zeiss OPMI PROergo, Carl Zeiss Surgical GmbH, Oberkochen, Germany). Canal bifurcation was carefully negotiated using a precurved #10 K-file (Dentsply Maillefer, Ballaigues, Switzerland) (Figure 2). The working length was assessed using a apex locator (Root ZX, J. Morita Co., Kyoto, Japan) and was later confirmed radiographically. Biomechanical preparation was done using ProTaper rotary files (Dentsply Maillefer, Ballaigues, Switzerland) till F2 for both the canals. Irrigation was accomplished using 5.25% sodium hypochlorite solution followed by 17% EDTA (Prevest Denpro Limited Digiana, Jammu, India). Saline was used as a final irrigant following which canal was dried using paper points (Dentsply Maillefer, Ballaigues, Switzerland). Fit of master cone was confirmed on radiograph. Apical portion of both the canals was obturated one by one using F2 ProTaper gutta percha (Dentsply Maillefer, Ballaigues, Switzerland) coated with a EndoSequence BC sealer (Brasseler, Savannah, GA, USA) with a System B device (SybronEndo, Orange, CA, USA). The rest of the canal was back filled using a Obtura gun (Obtura II, Spartan, Fenton, MO, USA).

**Figure 3:**
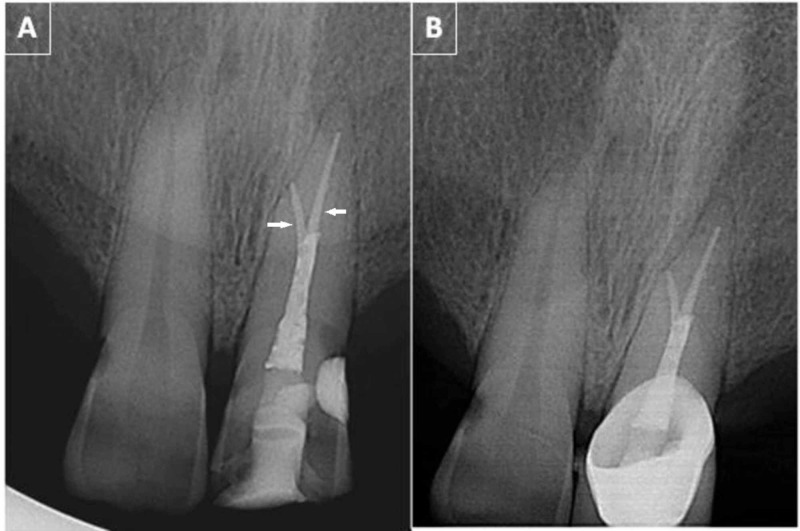
(A) Immediate postoperative radiograph of tooth number 21 showing well-obturated apical bifurcation (marked with arrows). (B) Twelve-month follow-up radiograph.

Access cavity was restored with a light-cured nanohybrid composite resin (Filtek Z250, 3M ESPE, St. Paul, MN) (Figure 3A). The patient was given full coverage porcelain fused to metal crown after two weeks. On follow-up appointment after 12 months, tooth was functional and the patient was asymptomatic. Radiograph also showed healing of periapical tissues (Figure 3B).

## Discussion

The objective of this paper was to present a case report illustrating the endodontic management of a maxillary central incisor with Vertucci’s type V root canal anatomy. To the author’s knowledge, only one case report has been published till date which shows type V root canal anatomy in maxillary central incisor [12]. No in vitro anatomic study has shown the presence of type V anatomy till date.

Vertucci’s type V root canal anatomy presents major endodontic challenge. To gain access to the apical third canal bifurcation, access opening needs to be carefully widened under the DOM to avoid root perforation. DOM is an indispensable armamentarium while managing complex root canal anatomy. It reduces the chances of mishaps, increases the chance of locating additional canals and offers greater precision [13]. Advanced imaging modalities like cone beam computed tomography (CBCT) can be particularly helpful in managing cases with complex morphology [14]. However, in the present case CBCT was not used as both the canals could be located and managed successfully under DOM. Careful inspection of pulp chamber walls and floor under proper illumination and magnification can assist in detecting accessory root canal.

Access cavity needs to be extended properly specially over the lingual shelf area to expose or uncover the second canal if present. Preoperative radiograph may indicate the presence of accessory root/root canal. A sudden break, narrowing or change in radiographic density of the root canal indicates presence of additional canal [15]. In the presented case report also sudden break was evident on the preoperative radiograph. In addition, the presence of radiolucency on lateral surface of root or two separate apical radiolucency also indicates towards additional canal [16].

## Conclusions

Although majority of studies suggest that maxillary central incisor is a single rooted tooth with single canal, a prudent clinician should always begin root canal procedure with an open mind and expect anatomical variations. It is highly recommended to use advanced armamentarium like DOM in managing teeth with complex root canal anatomy to provide much required precision in managing complex cases and increasing the ultimate success rate of endodontic treatment.
